# Efficient arbitrary simultaneously entangling gates on a trapped-ion quantum computer

**DOI:** 10.1038/s41467-020-16790-9

**Published:** 2020-06-11

**Authors:** Nikodem Grzesiak, Reinhold Blümel, Kenneth Wright, Kristin M. Beck, Neal C. Pisenti, Ming Li, Vandiver Chaplin, Jason M. Amini, Shantanu Debnath, Jwo-Sy Chen, Yunseong Nam

**Affiliations:** 1 IonQ, College Park, MD 20740 USA; 2 Wesleyan University, Middletown, CT 06459 USA

**Keywords:** Quantum information, Qubits

## Abstract

Efficiently entangling pairs of qubits is essential to fully harness the power of quantum computing. Here, we devise an exact protocol that simultaneously entangles arbitrary pairs of qubits on a trapped-ion quantum computer. The protocol requires classical computational resources polynomial in the system size, and very little overhead in the quantum control compared to a single-pair case. We demonstrate an exponential improvement in both classical and quantum resources over the current state of the art. We implement the protocol on a software-defined trapped-ion quantum computer, where we reconfigure the quantum computer architecture on demand. Our protocol may also be extended to a wide variety of other quantum computing platforms.

## Abstract (web summary)

Here, the authors devise an exact protocol that simultaneously entangles arbitrary pairs of qubits on a trapped-ion quantum computer. The protocol requires classical computational resources polynomial in the system size, and very little overhead in the quantum control compared to a single-pair case.

## Introduction

Quantum computers are expected to solve certain computational problems of interest more efficiently than classical computers using state-of-the-art classical algorithms. Notable examples include integer factorization^1^, unsorted database search^2^, and quantum dynamics simulations^3^. Multiple quantum computing platforms are under active development today. One of these platforms is the trapped-ion quantum information processor (TIQIP), which has demonstrated ^171^Yb^+^ qubit coherence times in excess of 10 minutes^4^, single-qubit gate fidelity of 99.9999%^5^, and two-qubit gate fidelity of 99.9%^6,7^. In addition, a TIQIP may leverage the all-to-all connectivity between ion qubits. The ability to directly apply a two-qubit gate to any pair of qubits provides TIQIPs an important advantage over other QIPs with limited connectivity^8^.

While the current progress in TIQIP technology is remarkable, better quality quantum gates are needed to run longer quantum programs and still obtain reliable quantum computational results^9^. The shortest quantum program known to date, expected to deliver scientifically meaningful discoveries, requires hundreds of thousands of quantum gates^10^. Therefore, to address quantum computational problems of broad interest, the two-qubit gate design in TIQIPs must be improved. An efficient procedure that simultaneously implements as many two-qubit gates as possible with the least amount of resources will thus accelerate the process of harnessing the power of universal, programmable quantum computers.

In this paper, we devise a new protocol that efficiently and simultaneously implements multiple two-qubit gates on a TIQIP. Using our efficient, arbitrary, simultaneously entangling (EASE) gates, arbitrary ion-qubit pairs, overlapping or not, can be entangled with programmable degrees of quantum entanglement. We implement EASE gates by modulating the amplitude of laser pulses that address individual ion qubits that comprise our scalable, general-purpose, programmable TIQIP, hosted at IonQ^11^. These new gates pave the way for efficient implementations of large-scale quantum algorithms on a TIQIP.

## Results

### Two-qubit gate on a trapped-ion quantum information processor

**Fig. 1:**
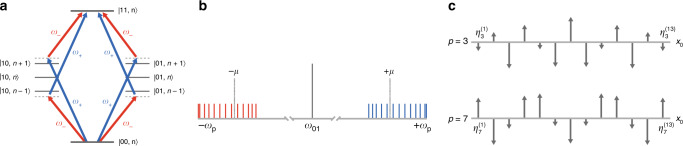
**Quantum dynamics of an EASE gate**. **a** An energy-level diagram associated with the *p*th motional mode showing off-resonant red and blue sideband transitions that cause the desired two-qubit coupling between $\left|00\right\rangle$ and $\left|11\right\rangle$ quantum states. Here, *ω*_±_ = *ω*_01_ ± *μ*, and *n* denotes the motional state. **b** Frequency spectrum of the motional modes *ω*_*p*_ of the ion chain centered around the carrier frequency *ω*_01_ that induces a single-qubit state transition. Symmetric detuning by frequency *μ* for red and blue sidebands is applied to the pulses that illuminate ions to induce the desired EASE gate. **c** Motional-mode diagrams that show the geometric structure of the modes. The ion displacements from their respective equilibrium positions are proportional to the coupling strength ${\eta }_{p}^{(m)}$ between the different ions *m* and the different modes *p*.

The native two-qubit gate on our TIQIP is implemented according to the Mølmer–Sørensen protocol^12–14^, which induces an effective XX-Ising interaction between a pair of qubits. The coupling between the computational states of the qubit pair is mediated by the motional modes of the linear *N*-ion chain stored in an ion trap. The evolution operator $\hat{U}$ that describes this operation is^15^

1 $$\hat{U}=\exp \left[\mathop{\sum }\nolimits_{m = 1}^{N}({\hat{\beta }}^{(m)}-{\hat{\beta }}^{(m)\dagger }){\hat{\sigma }}_{x}^{(m)}-i{\sum }_{n\ne m}{\chi }^{(m,n)}{\hat{\sigma }}_{x}^{(m)}{\hat{\sigma }}_{x}^{(n)}/4\right]$$

where ${\hat{\beta }}^{(m)}=i\mathop{\sum }\nolimits_{p = 1}^{N}{\alpha }_{p}^{(m)}(\tau ){\hat{a}}_{p}^{\dagger }$ (with motional-mode index *p*, coupling strength ${\alpha }_{p}^{(m)}$ between ion *m* and mode *p*, the *p*th motional-mode creation operator ${\hat{a}}_{p}^{\dagger }$—see Fig. 1—and the gate duration *τ*) denotes the coupling between the computational state of qubit *m* and the motional modes, ${\hat{\sigma }}_{x}^{(m)}$ is the Pauli-*x* operator on the *m*th qubit, and *χ*^(*m*, *n*)^ denotes the degree of entanglement between qubits *m* and *n*. To obtain a successful single-pair XX gate, we require that the first term in Eq. (1) and all *χ*^(*m*, *n*)^ vanish, except for *χ*^(*m*, *n*)^ of the targeted ion pair *m*, *n*. Similarly, to implement EASE gates between freely chosen pairs of qubits with an arbitrary degree of entanglement for every pair, we require that

(A) the first operator ${\hat{\beta }}^{(m)}$, which represents the coupling between motional modes of the ion chain and the computational states of the qubits, vanishes at the end of the evolution, and that
(B) the second operator’s coefficient *χ*^(*m*, *n*)^ either vanishes (if the ion pair *m*, *n* is not to be entangled) or computes to a pre-specified degree of entanglement (if the pair is to be entangled).

To satisfy conditions (A) and (B), we individually address participating ions with amplitude-modulated (AM) laser pulses^11^, where the modulation is performed by dividing the gate time *τ* into *N*_seg_ equi-spaced segments and allowing the amplitude to vary from one segment to the next.

Denoting the amplitude of the pulse Ω^(*m*)^(*t*) applied to ion *m* during segment *k* as ${\Omega }_{k}^{(m)}$, the laser detuning from the carrier frequency as *μ* and the motional-mode frequencies as *ω*_*p*_, condition (A) implies, for all *m* and *p*,

2 $${\alpha }_{p}^{(m)}(\tau ) =-{\eta }_{p}^{(m)}\int_{0}^{\tau }dt\ {\Omega }^{(m)}(t)\cos (\mu t){e}^{i{\omega }_{p}t}=0\\ \mapsto \sum_{k = 1}^{{N}_{{\rm{seg}}}}{\Omega }_{k}^{(m)}\int_{(k-1)\tau /{N}_{{\rm{seg}}}}^{k\tau /{N}_{{\rm{seg}}}}dt\ \cos (\mu t){e}^{i{\omega }_{p}t}=\hat{M}{{\boldsymbol{\Omega }}}^{(m)}=0,$$

where ${\eta }_{p}^{(m)}$ denotes the coupling constant (Lamb–Dicke parameter) for qubit *m* and mode *p* (see also Fig. 1), ${\hat{\boldsymbol{{M}}}}$ is the matrix with elements that are the segmented integrals shown above, and **Ω**^(*m*)^ is the vector of ${\Omega }_{k}^{(m)}$. Likewise, in the segmented form, condition (B) implies

3 $${\chi }^{(m,n)}=	\sum_{k = 1}^{{N}_{{\rm{seg}}}}\sum_{l = 1}^{k}{\Omega }_{k}^{(m)}{\Omega }_{l}^{(n)}\int_{(k-1)\tau /{N}_{{\rm{seg}}}}^{k\tau /{N}_{{\rm{seg}}}}d{t}_{2}\int_{(l-1)\tau /{N}_{{\rm{seg}}}}^{\min ({t}_{2},l\tau /{N}_{{\rm{seg}}})}d{t}_{1}\\ 	\left[-\sum_{p = 1}^{N}4{\eta }_{p}^{(m)}{\eta }_{p}^{(n)}\sin [{\omega }_{p}({t}_{2}-{t}_{1})]\cos (\mu {t}_{1})\cos (\mu {t}_{2})\right]\\ =	{({{\boldsymbol{\Omega }}}^{(m)})}^{T}\hat{\boldsymbol{{{D}}}}^{(m,n)}{{\boldsymbol{\Omega }}}^{(n)}\\ =	 \left\{\begin{array}{cc}{{\theta }^{(m,n)}} & {{\rm{if}}\,m\,{\rm{and}}\,n\,{\text{are}} \, {\text{to}} \,{\text{be}} \, {\text{entangled,}}} \\ 0 \hfill & {\rm{otherwise,}}\end{array}\right.$$

where ${\hat{\boldsymbol{{{D}}}}}^{(m,n)}={\hat{D}}^{(n,m)}$ is the triangular matrix with elements that are the segmented double integrals and the angle parameters ${\theta}^{(m, n)}$ denote the desired degree of entanglement between the qubit pair (*m*, *n*). We note that, according to Eq. (1), the desired evolution to be induced between qubits *m* and *n* is $\exp [-i({\chi }^{(m,n)}+{\chi }^{(n,m)}){\sigma }_{x}^{(m)}{\sigma }_{x}^{(n)}/4]$. Since the *χ*s are scalars, ${\chi }^{(m,n)}+{\chi }^{(n,m)}={\chi }^{(m,n)}+{({\chi }^{(m,n)})}^{T}$. Therefore, the constraint Eq. (3) may be rewritten as

4 $${\left({{\boldsymbol{\Omega }}}^{(m)}\right)}^{T}\hat{\boldsymbol{{{S}}}}^{(m,n)}{{\boldsymbol{\Omega }}}^{(n)} =\left\{\begin{array}{cc}{{\theta }^{(m,n)}} & {{\rm{if}}\,m\,{\rm{and}}\,n\,{\text{are}} \, {\text{to}} \,{\text{be}} \, {\text{entangled,}}} \\ 0 \hfill & {\rm{otherwise,}}\end{array}\right.$$

where ${\hat{\boldsymbol{{{S}}}}}^{(m,n)}=[{\hat{D}}^{(m,n)}+{({\hat{D}}^{(m,n)})}^{T}]/2$ is a symmetric matrix. The problem of finding the amplitude vectors **Ω** satisfying the two conditions Eq. (2) and Eq. (4) can, in principle, be written in the form of a quadratically constrained quadratic program (QCQP)^16^, which is in general NP-hard, as has been pointed out in the literature^17,18^. However, our problem is fully specified by the two equations, Eqs. (2) and (4), which is a special case of QCQP. The vectors **Ω** that satisfy Eq. (2) and Eq. (4) can be solved exactly in polynomial time using a linear approach.

### EASE-gate protocol

**Fig. 2:**
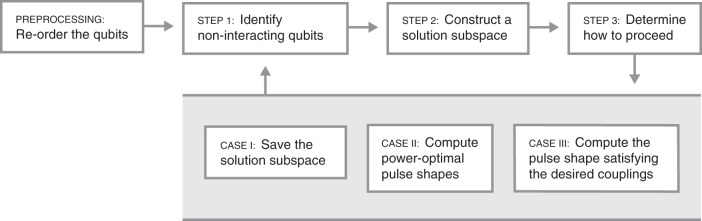
**Flowchart for EASE-gate pulse-shape synthesis**. As a preprocessing step, reorder those qubits that participate in a given EASE gate into disjoint, non-interacting sets, where the first and second qubits of each set interact. In other words, within each set, overlapping qubit pairs may interact, if the size of the set is larger than two. Consider now the following iterative steps. In Step 1, identify the qubits considered in the past iterations that do not interact with the currently considered qubit. In Step 2, construct a subspace orthogonal to the interactions between previously determined pulse shapes for qubits identified in Step 1 and the currently considered qubit. In Step 3, determine how to proceed based on the index of the currently considered qubit within its set. If that qubit is the first element of its set, proceed to Case I; if it is the second, proceed to Case II; otherwise, proceed to Case III. Case I: save the orthonormalized vectors spanning the subspace constructed in Step 2. Some linear combination of those vectors will yield a power-optimal pulse shape for the currently considered qubit. Case II: using two sets of orthonormal vectors, for the currently considered and the immediately preceding qubits, compute the power-optimal pulse shapes for those qubits given their interaction matrix. Case III: Compute the pulse shape for the currently considered qubit that satisfies the desired entangling interactions between itself and all of the previously considered qubits. We iterate Steps 1–3 until all participating qubits have been accounted for. This method may be used for non-overlapping or overlapping pairs of qubits. See Supplementary Note 2 for further details.

 Figure 2 shows a flowchart that outlines our linear approach to produce pulse shapes that implement an EASE gate. Once the experimental parameters, such as the number and positions of the ion qubits, the motional-mode frequencies of the ion chain, the Lamb–Dicke parameters, the detuning frequency, the desired EASE-gate duration, the number of AM segments, and the qubit pairs with corresponding degrees of entanglement are specified, our protocol constructs the $\hat{\boldsymbol{{M}}}$-matrix in Eq. (2). The null-space vectors of $\hat{\boldsymbol{{M}}}$ are then computed. They span a vector space from which we draw pulse shapes that satisfy Eq. (4).

To find a suitable pulse shape that requires minimal laser power, an important experimental concern, the $\hat{\boldsymbol{{S}}}$ matrix in Eq. (4) is first projected onto the null space of $\hat{\boldsymbol{{M}}}$. The eigenvector **c** with the largest absolute eigenvalue of the projected matrix is then guaranteed to require the minimal power possible, measured according to the sum of squares of the individual amplitudes ${\Omega }_{l}^{(m)}$. This methodology can then be iterated to find the pulse shapes for all ion qubits involved in the EASE gate (see Supplementary Notes 1 and 2 for theoretical details) by considering the pulse-shape search-space for a given qubit to be the intersection between the full null space and a subspace orthogonal to the space of previously identified pulse shapes for ions that the given qubit needs to be decoupled from.

We note that, even though an EASE gate with *N*_EASE_ participating qubits may require as many as *N*_EASE_(*N*_EASE_ − 1)/2 angle parameters *θ*^(*m*, *n*)^ (see Eq. (3)), we require only *N*_seg_ = 2*N* + *N*_EASE_ − 1 as the minimal number of segments, which is sufficient to satisfy all *χ*^(*m*, *n*)^ relations and ${\alpha }_{p}^{(m)}$ conditions. This is enabled by the fact that, for every additional participating qubit, we may start with the full set of null-space vectors that always satisfy condition (A), and the number of relations with respect to each of the participating qubits, according to condition (B), is at maximum *N*_EASE_ − 1. In other words, each participating qubit in an EASE gate is subject to at most 2*N* + *N*_EASE_ − 1 linear constraints. Because our approach is completely linear, the EASE-gate pulse shapes that exactly implement the desired operation are obtained in polynomial time.

### Implementation

We implement our EASE-gate protocol on a TIQIP hosted at IonQ^11^, which can load and control small chains of ^171^Yb^+^ ion qubits. Each qubit is optically initialized to a pure quantum state and then manipulated by addressing the qubit with pulses from a mode-locked 355-nm pulsed laser. These pulses can be engineered to drive either single-qubit operations by coupling to the internal (spin) degree of freedom of the ion, or two-qubit operations by coupling to both the internal and external (collective motional) degrees of freedom. We realize EASE gates by coupling the internal and external degrees of freedom of many ions simultaneously with segmented AM laser pulses.

**Fig. 3:**
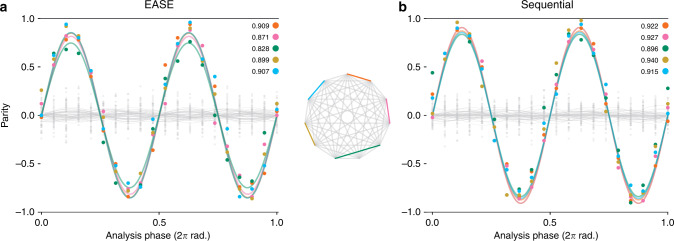
**Parity curves for EASE and sequential gates and a 11-qubit TIQIP all-to-all connectivity diagram**. The connectivity diagram displays the ion pairs used in the experiments. The associated fidelities are computed from the amplitudes of the measured parity and populations via maximum likelihood estimation. **a** Parity curve for an EASE gate with five simultaneous XX interactions. We chose pulses with *N*_seg_ = 35 and gate time *τ* = 924.0 μs. This gate yielded an average fidelity of $88.{3}_{-1.6}^{+1.0} \%$ with an average deviation from the ideal fidelity of $2.{3}_{-1.6}^{+2.1} \%$ for the 50 non-involved pairs. **b** Parity curve for a series of five sequential XX interactions. We chose pulses with *N*_seg_ = 27 for each XX gate with gate time *τ* = 318.6 μs, which yielded an average fidelity of $92.{0}_{-1.4}^{+0.8} \%$ with an average deviation from the ideal fidelity of $0.{9}_{-1.0}^{+2.4} \%$ for the 50 non-involved pairs. To ensure a fair comparison of gate times, we made sure that the peak powers at which we executed the EASE and the sequential gates differed by no more than 0.5%. Thus, comparing the EASE and sequential-based approaches to create the same final state of all qubits, application of the EASE gate saved ~669.0 μs, i.e., 42% of the total gate time needed in the sequential approach. The quoted errors are 1*σ* confidence intervals from the maximum likelihood estimation. See Supplementary Note 3 for implementation details.

In particular, we implemented EASE gates to fully entangle qubits in multiple disjoint pairs in a system with 11 ion qubits on a 13-ion chain. Of these qubits, up to 5 pairs (10 qubits) were simultaneously entangled. We then performed partial output state tomography on each entangled state by measuring the parity of the entangled pairs as a function of an analysis-pulse angle (shown in Fig. 3), and also measuring the even parity population without applying analysis pulses. By extracting the amplitude of the measured parity and populations via maximum likelihood estimation^7,11^, we are able to get a lower-bound estimate of the fidelity of the performed EASE gate. For our implementation with five simultaneous gates (Fig. 3a), we estimate an average gate fidelity of $F=88.{3}_{-1.0}^{+1.6} \%$. For the case in which we applied five gates sequentially (Fig. 3b), we estimate an average gate fidelity of $F=92.{0}_{-1.4}^{+0.8} \%$. The given errors on fidelity represent a 1*σ* confidence interval on the maximum likelihood estimation used to determine the fidelity.

We use the same technique to estimate any residual entanglement with non-addressed ions, due predominantly to optical crosstalk, by determining the overlap of any pair with the fully entangled Bell state we are trying to prepare. For pairs with one ion participating in a gate, the fidelity is ideally *F* = 25%, which corresponds to a fully mixed state. For pairs where neither ion participates in an applied gate, we expect to have *F* = 50% because the initial pure state has 50% overlap with the Bell state we are trying to prepare. The 50 non-involved pairs have $\delta F=2.{3}_{-1.6}^{+2.1} \%$ average deviation from the ideal fidelity for the five simultaneously applied gates (Fig. 3a). In the case of five sequentially applied gates (Fig. 3b), we see an average deviation from the ideal fidelity of $\delta F=0.{9}_{-1.0}^{+2.4} \%$. In these results, we have performed more simultaneous two-qubit entangling gates than previously reported^17^ on chains of ions at least twice as long as any previously reported results^17,18^. The fidelities reported here are markedly lower; however, it should be noted that our results are not corrected for state-preparation and measurement errors.

## Discussion

Because a TIQIP can induce couplings between arbitrary pairs of qubits by simply switching on and off pairwise interactions, the EASE gates developed and demonstrated here can readily be implemented on a TIQIP through software alone. This is in contrast to other quantum hardware platforms such as a solid-state QIPs, where each two-qubit interaction has to be hard-wired during the manufacturing process. TIQIPs can load as many qubits as necessary and employ the EASE-gate protocol to simultaneously implement any combinations of simultaneously addressible Ising interactions with little to no extra cost at the hardware level.

A host of quantum algorithms benefit from the ability to implement EASE gates. These algorithms tend to contain an orderly structure such that the circuit may be manipulated to reveal multiple Ising interactions applied simultaneously. For instance:

- Quantum arithmetic circuits^19,20^—useful for solving an integer factoring problem or computing discrete logarithms over Abelian groups^1^.
- Multi-control Toffoli gates using global XX gates as a special instance of an EASE gate^21^—useful, e.g., Grover’s unsorted database search algorithm^2^, applicable for solving certain satisfiability problems^9^.
- Fan-in or fan-out CNOTs or various roots of NOTs—useful for realizing the quantum Fourier transform^21^ or the Bernstein–Vazirani algorithm^22^.
- Disjoint *k*-local operators—useful for quantum simulation circuits, including both variational quantum eigensolver^23^ or Hamiltonian-dynamics simulations^10^, and the Hidden-shift algorithms^24^.

**Fig. 4:**
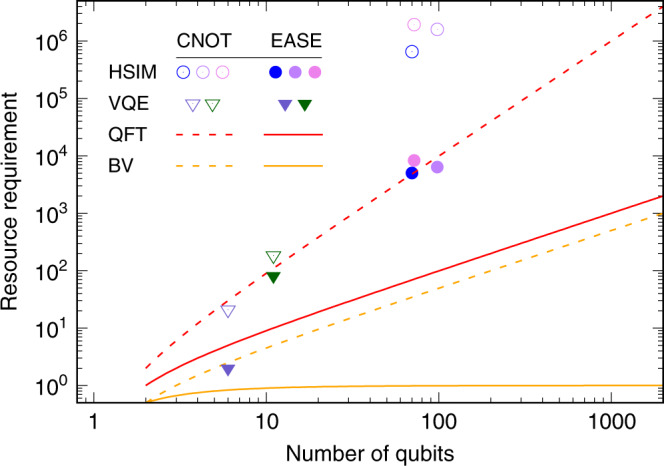
**Quantum resource requirement as a function of the number of qubits for various algorithms**. The resources are counted as the number of two-qubit CNOT gates for non-EASE-based implementation and multi-qubit EASE gates for EASE-based implementation. The quantum computational runtime or fidelity of the algorithms may vary in practice. See Fig. 3 for the details of the trade-off between the two approaches for the particular context described therein. Well-defined circuit layouts of the two-qubit entangling gates in all cases considered allow for negligible classical resource overhead in solving for EASE pulse shapes over the standard pairwise pulse shapes used in a serial approach. Shown are the Hamiltonian simulation (HSIM) algorithms (circles) simulating the Heisenberg Hamiltonian over various connectivity structures^10^, variational quantum eigensolver (VQE) circuits (triangles) simulating the water molecule with varying degrees of approximations^23^, quantum Fourier transform (QFT) circuits (red lines)^21^, and Bernstein–Vazirani (BV) algorithm (orange lines)^22^ with expected gate counts over all possible oracles of a fixed size. Hollow plot symbols and dashed lines denote the two-qubit CNOT gate based implementations. Solid plot symbols and solid lines denote the multi-qubit EASE-based implementations. Quadratic improvements in the resource requirement are observed for HSIM and QFT, and a linear to constant complexity improvement is observed for the BV and the Hidden-shift (not shown) algorithms. See Supplementary Note 4 for details on how to obtain EASE-gate counts.

To highlight the advantages offered by the EASE operation, in Fig. 4 we show a selection of notable algorithms that benefit from our efficient EASE-gate protocol.

Our EASE-gate protocol is linear and the pulse shapes we obtain exactly solve the problem and induce the desired quantum operation with up to *N*(*N* − 1)/2 angle parameters *θ*^(*m*, *n*)^ with minimal control overhead, i.e., linear in *N*, comparable to a single XX gate in terms of the number of segments. The shapes are generated in time polynomial in the system size and are power-optimal for the AM approach when used for a single XX gate with a fixed number of segments, since, in this case, the EASE protocol produces the pulse vector **Ω** with the minimal possible norm that implements a single entangling gate. This is in contrast to the non-linear, approximate methods used in previous studies^17,18^ that in general return an approximate pulse-shape solution and require an exponential overhead in the number of segments. Our protocol explains why it was possible in previous studies^17^ that a certain echo-based pulse-shape ansatz worked well for applying simultaneous gates on disjoint pairs of qubits—the shape automatically satisfies the entanglement requirement condition (B) and the infidelity owing to the imperfect decoupling from the motional modes, due to condition (A), may be minimized by navigating through the null space of $\hat{\boldsymbol{{M}}}$. Furthermore, our protocol enables us to entangle pairs of qubits with overlapping qubits.

Our protocol is scalable and is guaranteed to work for any modulation that admits a linear construction, such as the equi-spaced segment-based AM approach explored here or a more general approach demonstrated in ref. ^25^ (see Sec. S12 therein). In addition, because, again, our protocol admits a linear construction, we can readily take advantage of the high degree of stabilization with respect to external parameter fluctuations demonstrated in ref. ^25^ directly in the EASE-gate implementations, at the cost of additional degrees of freedom; in the segment-based AM method, this translates to an additional number of segments. The improved stability will likely lead to a better gate fidelity. Furthermore, we could leverage the first circuit identity that appears in Sec. IV of ref. ^21^ to remove certain crosstalk errors to first order: Note that the circuit identity reads XX(*φ*)(1 ⊗ *σ*_*z*_) XX(*φ*)(1 ⊗ *σ*_*z*_) = (1 ⊗ 1) for any entangling angle *φ*, where *σ*_*z*_ is the Pauli-*z* operator. As an example, this implies that a weak XX interaction or crosstalk, for instance induced between an EASE-participating qubit that sees the pulse shape **Ω** and a non-EASE-participating qubit that sits adjacent to one of the EASE-participating qubits that sees $\varepsilon {\boldsymbol{\Omega }}^{\prime}$, due to the spilled-over beam seen by the nonparticipating qubit, can be removed to first order in *ε* by repeating twice the interleaving of the *σ*_*z*_ gates on, e.g., a nonparticipating qubit and the EASE gate with half the desired entanglement angles. This costs, in our approach, a factor two in the number of segments used to implement an EASE gate. Our approach can be further generalized to mitigate higher order crosstalk errors at the cost of more segments.

We note that other quantum information processor architectures, such as those based on quantum dots^26^, neutral atoms^27,28^, or superconducting circuits^29,30^, also employ pulse-shape techniques to induce desired quantum operations. While the evolution operators for these approaches are not identical to the one considered here, the motivation behind the pulse shaping is the same: Remove the unwanted coupling while preserving the desired interaction from the architecturally-inducible Hamiltonian. We anticipate that the kind of efficient, linear approach we show here may be applicable for other qubit technologies with further research.

Classical supercomputers employ Multi-Instruction Multi-Data architectures and today’s personal computers typically employ Single-Instruction Multi-Data architectures. These parallel architectures have contributed significantly to sustaining the growth of classical processing power in the era where the frequency scaling of the processors has halted. Likewise, we expect the EASE protocol we explore in this paper to significantly boost the power of quantum computing, unlocking its ability to implement multiple entangling gates efficiently. Akin to the well-known Amdahl’s law in classical parallel computing^31^, we may roughly estimate the speed-up in quantum latency to scale inversely proportional to 1 − *p* + 2*pr*/*N*^2^, where *p* denotes the proportion of the quantum computational task that benefits from the simultaneous operations, *r* = *T*_EASE_/*T*_SINGLE_ denotes the latency overhead of an EASE gate with duration *T*_EASE_ over a single entangling gate with duration *T*_SINGLE_, and the factor *N*^2^/2 arises from the capability of the EASE gate to implement up to  ≈*N*^2^/2 entangling gates at a time. We believe simultaneously entangling gates, such as the EASE gates developed in this paper, will help ensure continued growth of the power of quantum processors, even when we encounter resource limitations per qubit.

## Supplementary information

Supplementary Information

## Data Availability

All data needed to evaluate the conclusions in the paper are present in the paper and/or the Supplementary Materials. Additional data related to this paper may be requested from the authors.

## Code availability

All necessary information to generate the code used to evaluate the conclusions in the paper are present in the paper and/or the Supplementary Materials.
