# Supplementary material for: Efficient arbitrary simultaneously entangling gates on a trapped-ion quantum computer
Source: Nat Commun. 2020 Jun 11;11:2963. doi: 10.1038/s41467-020-16790-9 (PMC7289877; doi:10.1038/s41467-020-16790-9)
Supplement: Supplementary file 1 — Supplementary Information [file 41467_2020_16790_MOESM1_ESM.pdf]

Efficient Arbitrary Simultaneously Entangling Gates on a Trapped-Ion Quantum Computer

Nikodem Grzesiak,[1,*] Reinhold Blümel,[1,2,†] Kenneth Wright,[1] Kristin M. Beck,[1] Neal C. Pisenti,[1] Ming Li,[1] Vandiver Chaplin,[1] Jason M. Amini,[1] Shantanu Debnath,[1] Jwo-Sy Chen,[1] and Yunseong Nam[1,‡]

[1]*IonQ, College Park, MD 20740, USA*

[2]*Wesleyan University, Middletown, CT 06459, USA*

Supplementary Note 1. SINGLE XX-GATE

As discussed thoroughly in the literature [1–5], the XX gate on an N-ion chain is implemented by ensuring the residual motion $\alpha_p^{(m)}$ for each ion m and motional mode p is zero, while the effective spin-spin interaction $\chi^{(m,n)}$ for the ion pair $(m,\ n)$ is non-zero. Specifically, in an amplitude-modulated implementation where we segment the pulse $\Omega^{(m)}(t)$ applied to the mth ion into $N_{\rm seg}$ equi-spaced segments, we require

$$\hat{M}\mathbf{\Omega}^{(m)} = \mathbf{0}, \tag{S1}$$

where the matrix elements of $\hat{M}$ are

$$\hat{M}_{p,k} = \int_{\tau_{k-1}}^{\tau_k} \cos(\mu t)\cos(\omega_p t)dt,$$
$$\hat{M}_{p+N,k} = \int_{\tau_{k-1}}^{\tau_k} \cos(\mu t)\sin(\omega_p t)dt, \tag{S2}$$

and

$$\mathbf{\Omega}^{(m)} = (\Omega_1^{(m)}\ \Omega_2^{(m)}\ ...\ \Omega_{N_{\rm seg}}^{(m)})^T, \tag{S3}$$

where $\Omega_k^{(m)}$, $k = 1, 2, ..., N_{\rm seg}$, is the signed amplitude of the kth segment, μ is the detuning from the carrier frequency, ω_p is the mode frequency, and $\tau_k = k\tau/N_{\rm seg}$. Additionally, we require

$$\chi^{(m,n)} = (\mathbf{\Omega}^{(n)})^T \hat{S}^{(m,n)} \mathbf{\Omega}^{(m)} \neq 0 \tag{S4}$$

with the symmetric matrix

$$\hat{S}^{(m,n)} = [\hat{D}^{(m,n)} + (\hat{D}^{(m,n)})^T]/2, \tag{S5}$$

where the matrix elements of the triangular matrix $\hat{D}^{(m,n)}$ are

$$\hat{D}_{k,l}^{(m,n)} = \int_{\tau_{k-1}}^{\tau_k} dt_2 \int_{\tau_{l-1}}^{\min(t_2,\tau_l)} dt_1 \left[-\sum_{p=1}^{N} 4\eta_p^{(m)}\eta_p^{(n)} \sin[\omega_p(t_2-t_1)]\cos(\mu t_1)\cos(\mu t_2)\right] \tag{S6}$$

and $\eta_p^{(m)}$ is the Lamb-Dicke parameter.

To satisfy conditions (S1) and (S4), we require $N_{\rm seg} > 2N$, such that there is at least one dimension available in the null-space of $\hat{M}$. We then linearly combine the orthonormal null-space vectors $\mathbf{\Omega}_{\rm null}^{[i]}$, where $i = 1, 2, ..., \mathcal{N}$, where $\mathcal{N}$ is the dimension of the null space of $\hat{M}$, to find a suitable vector $\mathbf{\Omega}^{(m)}$. We further assume that $\mathbf{\Omega}^{(m)} = \mathbf{\Omega}^{(n)}$, i.e., we illuminate the two qubits m and n targeted by the XX gate with the same pulses. Using now the $\mathcal{N}$ degrees of freedom, we may at this point optimize with respect to certain experimentally favorable conditions, such as the laser power. We found that, while the condition translates to finding the vector $\mathbf{\Omega}^{(m)}$ which minimizes $\max_l \Omega_l^{(m)}$, approximating this condition to finding the smallest sum of squares of $\Omega_l^{(m)}$ works well.

Specifically, to find the approximate solution to the power minimization requirement, we first reduce the solution space characterized by $\hat{S}^{(m,n)}$ to within the null space of $\hat{M}$. We do this by conjugating $\hat{S}^{(m,n)}$ with the orthonormal null-space vectors $\mathbf{\Omega}_{\rm null}^{[i]}$ for $i = 1, 2, .., \mathcal{N}$, i.e., we construct the reduced matrix $\hat{V}^{(m,n)}$ with matrix elements

$$\hat{V}_{i,j}^{(m,n)} = (\mathbf{\Omega}_{\rm null}^{[i]})^T \hat{S}^{(m,n)} \mathbf{\Omega}_{\rm null}^{[j]}. \tag{S7}$$

We then find the normalized eigenvector $\mathbf{c}$ of $\hat{V}^{(m,n)}$ with the largest absolute eigenvalue λ . The eigenvector $\mathbf{c}$ may now be used to find the desired pulse:

$$\mathbf{\Omega}^{(m)} = \mathbf{\Omega}^{(n)} = \left|\frac{\theta^{(m,n)}}{\lambda}\right|^{1/2} \sum_{i=1}^{\mathcal{N}} c_i \mathbf{\Omega}_{\rm null}^{[i]}. \tag{S8}$$

Supplementary Note 2. EASE GATE

To find pulses that entangle multiple pairs of ions simultaneously, we once again start with the full set of null-space vectors $\mathbf{\Omega}_{\rm null}^{[i]}$, $i = 1, 2, .., \mathcal{N}$. These vectors, by construction, automatically decouple the spin and motional states and satisfy the condition stated in (S1). Therefore, for any qubits that participate in a given EASE gate, the search for a suitable pulse starts from the null space spanned by $\mathbf{\Omega}_{\rm null}^{[i]}$.

Consider now an EASE gate that operates on $N_{\rm EASE}$ qubits. As a preprocessing step, we first perform the following:

*Electronic address: grzesiak@ionq.co
†Electronic address: blumel@ionq.co
‡Electronic address: nam@ionq.co

Preprocessing – Reorder the qubit indices such that disjoint sets of qubits are grouped and labeled consecutively; in a graph constructed with vertices that denote each qubits and edges that denote constituent XX gates of a given EASE gate with non-zero degree of entanglement, a pair of qubits may be considered disconnected if there is no path in this graph that has the two qubits as endpoints. A collection of connected qubits form a disjoint set. In particular, the ordering is done for each of the disjoint sets in such a way that the first and second element of each set are directly connected, i.e., there is an edge that connects the two qubits.

We now find a suitable pulse for each of the $N_{\rm EASE}$ participating qubits. The following procedure may then be iterated for each ion to obtain the desired pulse.

Step 1 – For the nth qubit, identify the desired XX interaction strengths between the previous $n{-}1$ qubits and the current nth qubit. Then, collect those interactions whose strengths are zero, i.e., determine which of the $n{-}1$ qubits are not to be coupled with the nth qubit.

Step 2 – From the full null space spanned by $\mathbf{\Omega}^{[i]}_{\rm null}$, construct a subspace that is orthogonal to the vectors $\hat{S}^{(m,n)}\mathbf{\Omega}^{(m)}$, where $m{<}n$ are the qubit indices of those qubits not coupled to the nth qubit, identified in Step 1.

Step 3 – Determine which of the following three cases are applicable. Case I – No qubits $m{<}n$ are coupled to the nth qubit. This case occurs for every first qubit in a distinct disjoint set. Case II – Some qubits $m{<}n$ are to be coupled to the nth qubit, but one of them, e.g., qubit index $n{-}1$, has a yet-to-be-determined pulse shape. This case occurs for every second qubit in a distinct disjoint set. Case III - Some qubits $m{<}n$ are to be coupled to the nth qubit and the pulse shapes for all of those qubits are already determined. This case occurs for the third qubit and onward in a distinct disjoint set.

The three cases I–III determine which procedure we select as the final step of the iteration before the start of another iteration. In particular,

- Case I – Save the orthonormal vectors $\mathbf{v}^{(n)}_l$, $l = 1,2,...,\mathcal{N}{-}(n{-}1)$, that span the subspace identified in Step 2. Continue to qubit number $n{+}1$.

- Case II – Compute orthonormal vectors $\mathbf{v}^{(n)}_l$, $l = 1,2,...,\mathcal{N}-(n-2)$, that span the subspace identified in Step 2. Also compute $\hat{V}^{(n-1,n)}_{i,j} = (\mathbf{\Omega}^{[i]}_{\rm null})^T\hat{S}^{(n-1,n)}\mathbf{\Omega}^{[j]}_{\rm null}$ and find the eigenvector $\mathbf{c}$ with the largest absolute eigenvalue. The desired pulse shape solutions for qubit $n{-}1$ and n are the closest pulse shapes that can be generated from their respective pulse-shape search-space to $\sum_i c_i\mathbf{\Omega}^{[i]}_{\rm null}$. Note that the vectors $\mathbf{v}^{(n-1)}$ span the pulse-shape search-space for the $n{-}1$st qubit. Once the pulse shapes for both qubits, $n{-}1$ and n, are determined, continue to qubit number $n{+}1$.

- Case III – Restarting from Step 1, i.e., with all of the null-space vectors $\mathbf{\Omega}^{[i]}_{\rm null}$, compute the basis vectors that span the space, in which the inner-product relations with $\hat{S}^{(m,n)}\mathbf{\Omega}^{(m)}$ for all qubits $m{<}n$ are satisfied. The inner products here denote the coupling strengths. The pulse shape for the nth ion is an appropriate combination of the basis vectors such that the norm is minimized. Continue to qubit number $n{+}1$.

We next detail the computational steps used to address each case. For case I, we need to find orthonormal vectors $\mathbf{v}^{(n)}$ drawn from the full null space spanned by $\mathbf{\Omega}^{[i]}_{\rm null}$ such that they are orthogonal to $\hat{S}^{(m,n)}\mathbf{\Omega}^{(m)}$, $m = 1,2,..,n{-}1$. Therefore, we require that

$$(\mathbf{\Omega}^{(m)})^T\hat{S}^{(m,n)}\mathbf{\Omega}^{(n)} = (\hat{V}^{(m,n)}\xi^{(m)})^T\zeta^{(n)} = 0, \qquad \text{(S9)}$$

where we used $\hat{V}^{(m,n)}$ in (S7), $\mathbf{\Omega}^{(m)} = \sum_i \xi^{(m)}_i\mathbf{\Omega}^{[i]}_{\rm null}$, and $\mathbf{\Omega}^{(n)} = \sum_i \zeta^{(n)}_i\mathbf{\Omega}^{[i]}_{\rm null}$. To find $\zeta^{(n)}$, we define a matrix $\hat{\epsilon}$ whose rows are $(\hat{V}^{(m,n)}\xi^{(m)})^T$. We then obtain $\hat{\tilde{\epsilon}}\zeta^{(n)} = \mathbf{0}$, where $\hat{\tilde{\epsilon}}$ is the row-reduced $\hat{\epsilon}$. This means that

$$\zeta^{(n)}_{j'} = -\sum_{j=n}^{\mathcal{N}}\tilde{\epsilon}_{j',j}\zeta^{(n)}_j \qquad \text{(S10)}$$

for $j' = 1,2,..,n-1$, and thus the desired $\zeta^{(n)}$-space is spanned by vectors ρ_l, where

$$\rho_l \in \left\{ \begin{pmatrix} -\tilde{\epsilon}_{1,n} \\ -\tilde{\epsilon}_{2,n} \\ \vdots \\ -\tilde{\epsilon}_{n-1,n} \\ 1 \\ 0 \\ 0 \\ \vdots \\ 0 \end{pmatrix}, \begin{pmatrix} -\tilde{\epsilon}_{1,n+1} \\ -\tilde{\epsilon}_{2,n+1} \\ \vdots \\ -\tilde{\epsilon}_{n-1,n+1} \\ 0 \\ 1 \\ 0 \\ \vdots \\ 0 \end{pmatrix}, \cdots, \begin{pmatrix} -\tilde{\epsilon}_{1,\mathcal{N}} \\ -\tilde{\epsilon}_{2,\mathcal{N}} \\ \vdots \\ -\tilde{\epsilon}_{n-1,\mathcal{N}} \\ 0 \\ 0 \\ 0 \\ \vdots \\ 1 \end{pmatrix} \right\}. \qquad \text{(S11)}$$

To this end, we obtain $\mathbf{v}^{(n)}_l$ in Case I to be $\sum_i(\varrho_l)_i\mathbf{\Omega}^{[i]}_{\rm null}$, where ϱ_l are the orthonormalized ρ_l in (S11).

For case II, we start with

$$\begin{aligned}
&(\mathbf{\Omega}^{(n-1)})^T\hat{S}^{(n-1,n)}\mathbf{\Omega}^{(n)} \\
&= \left[\sum_{l=1}^{n-2} b_l\left(\sum_{i=1}^{\mathcal{N}}\left(\varrho^{(n-1)}_l\right)_i\mathbf{\Omega}^{[i]}_{\rm null}\right)\right]^T \\
&\quad \hat{S}^{(n-1,n)}\left[\sum_{l'=1}^{n-2} b'_{l'}\left(\sum_{i'=1}^{\mathcal{N}}\left(\varrho^{(n)}_{l'}\right)_{i'}\mathbf{\Omega}^{[i']}_{\rm null}\right)\right] \\
&= \left[\sum_{i=1}^{\mathcal{N}}\left(\sum_{l=1}^{n-2} b_l\left(\varrho^{(n-1)}_l\right)_i\right)\mathbf{\Omega}^{[i]}_{\rm null}\right]^T \\
&\quad \hat{S}^{(n-1,n)}\left[\sum_{i'=1}^{\mathcal{N}}\left(\sum_{l'=1}^{n-2} b'_{l'}\left(\varrho^{(n)}_{l'}\right)_{i'}\right)\mathbf{\Omega}^{[i']}_{\rm null}\right] \\
&= \mathbf{B}^T\hat{V}^{(n-1,n)}\mathbf{B}', \qquad \text{(S12)}
\end{aligned}$$

where $B_i = \sum_l b_l(\varrho^{(n-1)}_l)_i$ and $B'_{i'} = \sum_{l'} b'_{l'}(\varrho^{(n)}_{l'})_{i'}$. Denoting the eigenvector of $\hat{V}$ with the largest absolute

eigenvalue as $\mathbf{L}$, we aim to find $\mathbf{B}$ and $\mathbf{B}'$ that have the largest overlap with $\mathbf{L}$. We obtain the suitable choices of b_l by computing the inner product between $\varrho_l^{(n-1)}$ and $\mathbf{L}$, and likewise for $b'_{l'}$; in particular, $b_l = \varrho_l^{(n-1)} \cdot \mathbf{L}/A$ and $b'_{l'} = \varrho_{l'}^{(n)} \cdot \mathbf{L}/A'$, where the normalization constants A and A' are chosen such that $\mathbf{B}^T \hat{V}^{(n-1,n)} \mathbf{B}' = \theta^{(n-1,n)}$.

For case III, we aim to find $\mathbf{\Omega}^{(n)}$ such that the main text Eq. (4) is satisfied. To do so, we start with all of the null-space vectors $\Omega_{\text{null}}^{[i]}$. Then, we require that

$$(\mathbf{\Omega}^{(m)})^T \hat{S}^{(m,n)} \mathbf{\Omega}^{(n)} = (\hat{V}^{(m,n)} \xi^{(m)})^T \zeta^{(n)} = \chi^{(m,n)}, \tag{S13}$$

where $\chi^{(m,n)}$ denotes the degree of entanglement between qubits m and n, $m<n$, $\hat{V}^{(m,n)}$ is defined in (S7), $\mathbf{\Omega}^{(m)} = \sum_i \xi_i^{(m)} \mathbf{\Omega}_{\text{null}}^{[i]}$, and $\mathbf{\Omega}^{(n)} = \sum_i \zeta_i^{(n)} \mathbf{\Omega}_{\text{null}}^{[i]}$. To find $\zeta^{(n)}$, we once again define a matrix $\hat{\epsilon}$ whose rows are $(\hat{V}^{(m,n)} \xi^{(m)})^T$. We then obtain $\hat{\tilde{\epsilon}} \zeta^{(n)} = \tilde{\chi}^{(n)}$, where $\hat{\tilde{\epsilon}}$ is the row-reduced $\hat{\epsilon}$ and the vector elements $\tilde{\chi}_m^{(n)}$ are the accordingly row-operated $\chi^{(m,n)}$ values. Then, the subspace that satisfies the aforementioned inner-product conditions is spanned by

$$(\rho_l)_i = \begin{cases} \tilde{\chi}_i^{(n)} - h_{n+l-1} \tilde{\epsilon}_{i,n+l-1} & \text{if } i < n, \\ h_{n+l-1} & \text{if } i = n+l-1, \\ 0 & \text{otherwise,} \end{cases} \tag{S14}$$

where h_{n+l-1} are free parameters and $l = 1, 2, ..., \mathcal{N} - (n-1)$.

We now ought to find appropriate coefficients r_l such that $\zeta^{(n)} = \sum_l r_l \rho_l$, where $\sum_l r_l = 1$, and the norm $|\zeta^{(n)}|$ is minimized. This amounts to solving $\partial |\zeta^{(n)}| / \partial d_l = 0$, where $d_l = h_{n+l-1} r_l$. It can be shown straightforwardly that in matrix form this may be expressed as

$$\mathbf{d} = \hat{P}^{-1} \phi, \tag{S15}$$

where the matrix elements of $\hat{P}$ are

$$\hat{P}_{l,l'} = \begin{cases} 1 + \sum_{q=1}^{n-1} (\tilde{\epsilon}_{q,n+l-1})^2 & \text{if } l = l', \\ \sum_{q=1}^{n-1} \tilde{\epsilon}_{q,n+l-1} \tilde{\epsilon}_{q,n+l'-1} & \text{if } l \neq l', \end{cases} \tag{S16}$$

where $l, l' = 1, 2, .., \mathcal{N} - (n-1)$, and the vector elements of ϕ are

$$\phi_l = \sum_{q=1}^{n-1} \tilde{\epsilon}_{q,n+l-1} \tilde{\chi}_q^{(n)}. \tag{S17}$$

The desired ζ is thus

$$\zeta_i = \begin{cases} \tilde{\chi}_i^{(n)} - \sum_{l=1}^{\mathcal{N}-(n-1)} \tilde{\epsilon}_{i,n+l-1} d_l, & \text{if } i < n, \\ d_{i-n+1}, & \text{if } i \geq n. \end{cases} \tag{S18}$$

Supplementary Note 3. IMPLEMENTATION DETAILS

Mode Frequencies (MHz)
2.698
2.704
2.721
2.739
2.758
2.779
2.800
2.820
2.840
2.858
2.874
2.890
2.895

TABLE S1: Mode frequencies of the motional modes of our 13-ion chain for the EASE gate shown in Fig. S1.

Mode Frequencies (MHz)
2.704
2.710
2.727
2.745
2.764
2.785
2.806
2.827
2.846
2.865
2.881
2.896
2.901

TABLE S2: Mode frequencies of the motional modes of our 13-ion chain for the XX gates shown in Fig. S1.

In this section, we detail the pulse-level implementation details. Figures S1 and S2 show the pulse shapes used to implement the five XX gates on our 11-qubit, 13-ion TIQIP. Commensurate to the protocol, we use $2N + N_{\text{EASE}} - 1 = 35$ segments for the five pairs of XX interactions for the EASE ($N = 13$ and $N_{\text{EASE}} = 10$) and $2N + N_{\text{EASE}} - 1 = 27$ for each of the five XX interactions used in the serial approach ($N = 13$ and $N_{\text{EASE}} = 2$ for each gate). The mode frequencies for each cases are reported in Tables S1 and S2, for EASE and sequential based approaches, respectively. The detuning frequencies μ_{EASE}, used for the EASE gate, is $\mu_{\text{EASE}} = 2.7884$ MHz, which may be compared to the frequencies $\mu_{i,j}$, used for the sequential based approaches between qubits i and j, $\mu_{0,1} = 2.768$ MHz, $\mu_{2,3} = 2.768$ MHz, $\mu_{4,6} = 2.7485$ MHz, $\mu_{7,8} = 2.768$ MHz, $\mu_{9,10} = 2.768$ MHz.

To produce the parity curves in the main text Fig. 3,we implemented a phase-stabilized version of the XX gates. Specifically, for each phase-sensitive XX gate, we applied $R_y(-\pi/2) = e^{-i(-\pi/2)\sigma_y/2}$ and its complex conjugate before and after the gate, respectively, on the qubits that participate in the XX gate. The single-qubit R_y gates here are implemented in the phase-sensitive configuration. The combined gate is a ZZ gate, known to be in-

sensitive to phase [6].

To now produce a phase-insensitive XX gate, we conjugate the ZZ gate with $R_y(\pi/2) = e^{-i(\pi/2)\sigma_y/2}$ and its complex conjugate before and after the ZZ gate. The single qubit gates R_y's here are implemented in the phase-insensitive configuration. The combined gate is the phase-stabilized XX gate.

The above method straightforwardly generalizes to the EASE gates. Since the parity curve characterizes the XX gates and we implement phase-insensitive version of XX gates, no change in the quantum circuit that needs to be run to produce the parity curves as shown in the main text Fig. 3is required.

Supplementary Note 4. EASE GATE COUNTS

We detail in this section the methods used to compute the EASE gate counts shown in Fig. 4 of the main text. The readers are strongly encouraged to read the corresponding references cited herein for each of the following considered cases. The cases considered here are (i) Heisenberg Hamiltonian simulation circuits over various connectivity patterns [7], (ii) water molecule simulation, using a variational eigensolver with varying degrees of approximation [8], (iii) quantum Fourier transform [9], (iv) Bernstein-Vazirani algorithms [10], averaged over all possible oracles of a given size, and (v) Hidden-Shift algorithms [11] with inner-product function.

We used the Heisenberg-Hamiltonian simulation circuits in [7] with fourth-order product formulas as benchmarks. Specifically, the considered connectivity graphs are (3−5−70), (4−4−98), and (5−3−72), where $(k{-}d{-}n)$ is a graph with degree k, diameter d, and number of vertices n. The CNOT gate counts are reported in Table I of [7]. The EASE gate counts are computed by the following procedure.

We note that the Heisenberg Hamiltonian over two qubits is of the form $\sigma_x\sigma_x + \sigma_y\sigma_y + \sigma_z\sigma_z$. Therefore, we may order the Hamiltonian terms such that all of the $\sigma_x\sigma_x$ terms appear consecutively, and likewise for $\sigma_y\sigma_y$ and $\sigma_z\sigma_z$. In this case, each stage of the fourth-order product-formula-based approximation of the evolution operator with the reordered Hamiltonian can be shown to have 30 sets of $\sigma_x\sigma_x$, $\sigma_y\sigma_y$, or $\sigma_z\sigma_z$ interactions, 9 of which can be merged since a $\sigma_x\sigma_x$ interaction followed by another $\sigma_x\sigma_x$ interaction is nothing but the combined $\sigma_x\sigma_x$ interaction. Therefore, there are a total of 21 $\sigma_i\sigma_i$, $i \in \{x, y, z\}$ interaction sets per product-formula stage. We recall that the EASE gate can implement each interaction set simultaneously. Thus, the total number of EASE gates evaluates to the number of product-formula stages times 21. The number of stages required may be found in Equation (4) of [7].

For the water molecule simulations, we considered HF+7 and HF+21 cases; HF stands for Hartree-Fock as defined in [8]. The HF+7 case is the pairwise excitation, bosonic-only case with 7 such terms, where the effective evolution operator is expanded using the first-order product formula. This case can be shown to require 14 XX gates (two XX gates with continuous parameters per excitation), or 21 CNOT gates, since any unitary operation over two qubits does not require more than three CNOT gates [12]. For the EASE-based approach, we reorder the excitation operators such that each one of the two XX gates per excitation can be grouped together to then be amenable to implementation by a single EASE gate. This results in two EASE gates in total for the HF+7 case. For the remaining case of HF+21, with the three-CNOT implementation above, the CNOT count is 185. For the EASE-gate consideration, we allow for parallel implementation of excitations over disjoint sets of qubits. Careful arrangement of the excitation terms results in 81 EASE gates in total.

The n-qubit quantum Fourier transform requires $n(n-1)$ CNOT gates, where we used two CNOT gates per controlled-z^a gate, where z^a is defined according to

$$z^a := \begin{bmatrix} 1 & 0 \\ 0 & e^{i\pi a} \end{bmatrix}. \tag{S19}$$

With EASE gates, each of the $n{-}1$ layers of controlled-z^a rotations, $a \in \{1/2, 1/4, ..., 1/2^l\}$, where l is the layer number, can be implemented simultaneously. Therefore, the EASE-gate counts for the quantum Fourier transform is $n{-}1$.

The Bernstein-Vazirani algorithm [10], implemented on an n-qubit quantum computer has an oracle size of $n-1$. Therefore, on average, $(n{-}1)/2$ CNOT gates are required to implement the oracle. The EASE gate allows for the implementation of any non-zero bit-string oracle in a single operation.

The Hidden Shift algorithm with inner-product function [11], implemented on a n-qubit (n even) quantum computer, requires n CNOT gates. Since this circuit requires two layers of parallel CNOT gates, each with $n/2$ CNOT gates, the EASE protocol allows us to implement the n-qubit Hidden-Shift algorithm with only two operations regardless of the number of qubits.

[1] C. Figgatt, A. Ostrander, N. M. Linke, K. A. Landsman, D. Zhu, D. Maslov, C. Monroe, Parallel entangling operations on a universal ion trap quantum computer. *Nature* **572**, 368–372 (2019).

[2] Y. Lu, S. Zhang, K. Zhang, W. Chen, Y. Shen, J. Zhang, J.-N. Zhang, K. Kim, Scalable global entangling gates on arbitrary ion qubits. *Nature* **572**, 363–367 (2019).

[3] S.-L. Zhu, C. Monroe, L.-M. Duan, Arbitrary-speed

quantum gates within large ion crystals through minimum control of laser beams. *Europhys. Lett.* **73**, 485–491 (2006).

[4] S. Debnath, N. M. Linke, C. Figgatt, K. A. Landsman, K. Wright, C. Monroe, Demonstration of a small programmable quantum computer with atomic qubits. *Nature* **536**, 63–66 (2016).

[5] P. H. Leung, K. A. Landsman, C. Figgatt, N. M. Linke, C. Monroe, K. R. Brown, Robust 2-qubit gates in a linear ion crystal using a frequency-modulated driving force. *Phys. Rev. Lett.* **120**, 020501 (2018).

[6] T. R. Tan, J. P. Gaebler, Y. Lin, Y. Wan, R. Bowler, D. Leibfried, D. J. Wineland Multi-element logic gates for trapped-ion qubits. *Nature* **528**, 380–383 (2015).

[7] Y. Nam, D. Maslov, Low cost quantum circuits for classically intractable instances of the Hamiltonian dynamics simulation problem. *npj Quant. Inf.* **5**, 44 (2019).

[8] Y. Nam *et al.*, Ground-state energy estimation of the water molecule on a trapped ion quantum computer. *npj Quant. Inf.* **6**, 33 (2020).

[9] D. Maslov, Y. Nam, Use of global interactions in efficient quantum circuit constructions. *New J. Phys.* **20**, 033018 (2018).

[10] E. Bernstein, U. Vazirani, Quantum complexity theory *SIAM J. Comput.* **26**, 1411–1473 (1997).

[11] W. van Dam, S. Hallgren, L. Ip, Quantum algorithms for some hidden shift problems. *SIAM J. Comput.* **36**, 763–778 (2006).

[12] V. V. Shende, I. L. Markov, S. S. Bullock, Minimal universal two-qubit controlled-NOT-based circuits. *Phys. Rev. A* **69**, 062321 (2004).

EASE Implementation

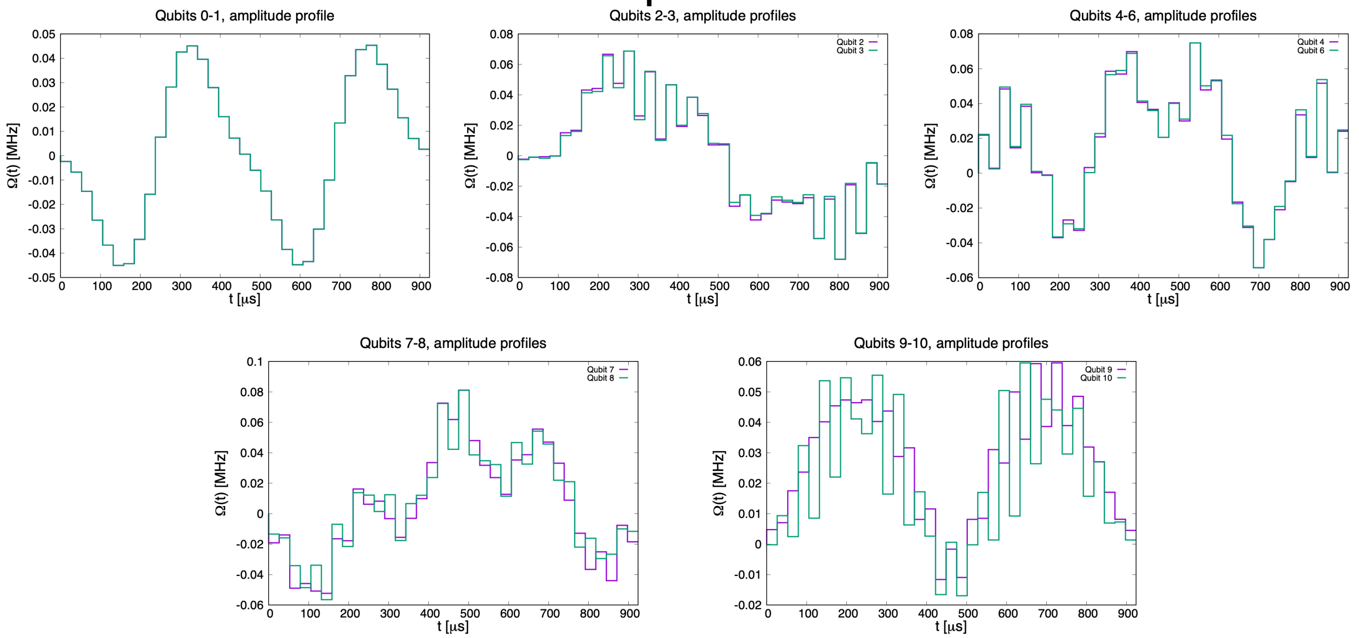

FIG. S1: Pulse shapes used in the EASE implementation of many two-qubit entantgling gates. The gate time of each of the 5 pulses is τ=924.0μs.

Sequential Implementation

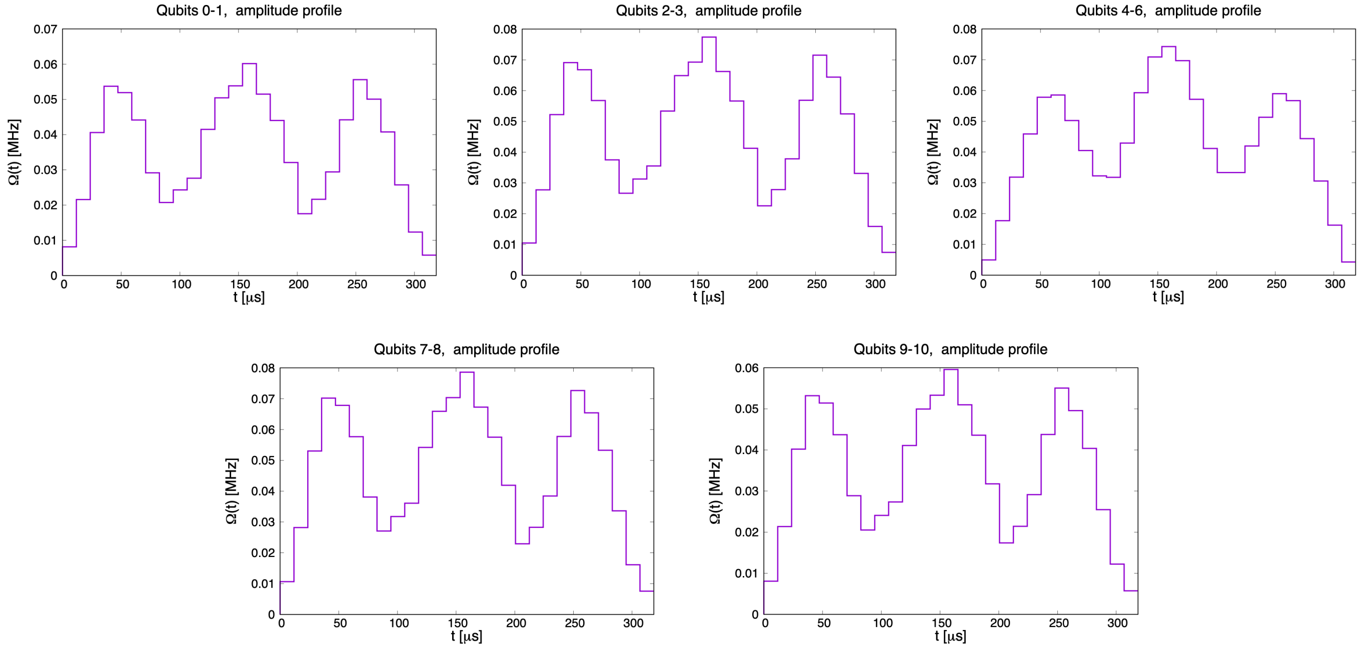

FIG. S2: Pulse shapes used in the sequential implementation of many two-qubit entantgling gates. The gate time of each of the 5 pulses is τ=318.6μs.
